# Relation of giant right atrial mass and thrombocytopenia; Recovery with surgery

**DOI:** 10.34172/jcvtr.025.32978

**Published:** 2025-03-18

**Authors:** Roghayeh Pourkia, Sadegh Sedaghat, Hamid Reza Vafaei, Seyed Sina Taheri Otaghsare

**Affiliations:** ^1^Department of Cardiology, School of Medicine, Rouhani Hospital, Babol University of Medical Sciences, Babol, Iran; ^2^Department of Internal Medicine, School of Medicine, Cancer Research Center, Health Research Institute, Rouhani Hospital, Babol University of Medical Sciences, Babol, Iran; ^3^Department of Surgery, School of Medicine, Rouhani Hospital, Babol University of Medical Sciences, Babol, Iran; ^4^Clinical Research Development Unit of Rouhani Hospital, Babol University of Medical Sciences, Babol, Iran

**Keywords:** Thrombocytopenia, Atrial myxoma, Polycythemia

## Abstract

As we know, cardiac myxoma is one of the most primary cardiac masses but laboratory findings in this type of tumor is non-specific and the diagnosis is by imaging. In this case we have reported a 61 year old man came to the emergency ward of hospital with history of recent onset dyspnea and The Laboratory finding indicates polycythemia with thrombocytopenia. Ultrasonography of abdomen and bone marrow aspiration and biopsy revealed no significant diagnosis but on echocardiography a large sized mass was detected in right atrium. After cardiologist and cardiac surgeon consultation the plan was to surgical Excision and after that the thrombocytopenia has been resolved. In this case report we want to write about a rare correlation between cardiac myxoma and thrombocytopenia and show that early diagnosis and treatment of the disease can help and totally cure complaints of patient.

## Introduction

 Primary cardiac tumors are rare but the most common one is cardiac myxoma arising from arterial chambers. The symptoms range from asymptomatic patient to sudden serious cardiopulmonary collapse.

 Because of that early diagnosis and treatment is important. Echocardiography even transthoracic or trans-esophageal is still the gold standard imaging for diagnosis and follow-up the patient.in this case report a 61 year old man admitted to emergency Ward with new-onset history of positional dyspnea and episodes of loss of consciousness.

## Case Report

 A 61-year-old man admitted to the hospital with new onset history of non-exertional dyspnea that becomes exaggerated in sitting and upright position and get better in supine position.

 The symptoms started from approximately 3 weeks ago.

 He complained even of brief episodes of sudden loss of consciousness in sitting position on history. There was no complaints of chest pain or diaphoresis.

 No recent loss of weight. He was diabetic and smoker but didn’t have any previous history of known cardiovascular disorder.

 EKG revealed normal sinus rhythm with poor precordial R-R progression and mild ST depression in inferior leads.

## Results

 On lab data the positive finding was thrombocytopenia (54000 at first blood sample that was decreased to 15000/mm3) and the hemoglobin level was 19.6 g/dl. Renal and hepatic function were normal. CRP level was upper limit of normal range. LDH level was normal. On PBS low platelet level was confirmed and they were Markley decreased.

 Emergency Para clinic imaging was requested and pulmonary CT angiography revealed no sign of pulmonary embolism. Abdominal sonography was negative for any organomegaly.

 Because of thrombocytopenia and polycythemia, patient was admitted to hematology service and cardiology consults requested for evaluation of dyspnea.

 On Bone marrow aspiration and biopsy megakaryocytes were within normal count and morphology and plasma cells were less than 2% of hematopoietic cells and all data were consistent with near normocellular marrow.

**Figure 1:**
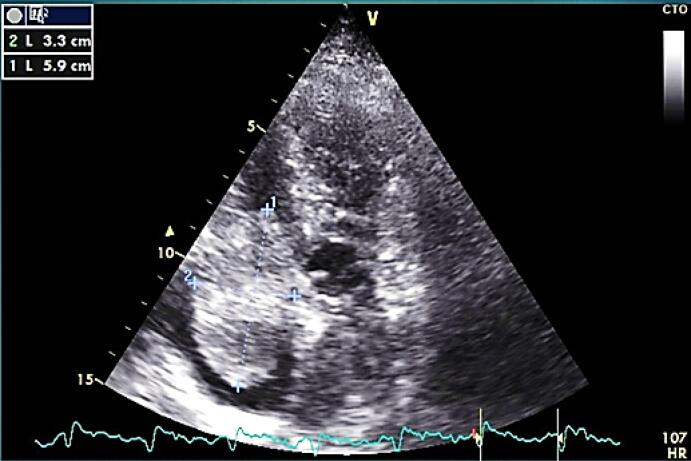
large size round shape mass in RA on TTE

 On transthoracic echocardiography a large size mobile pedunculated round-shape mass (5.9cm*3.3cm) in right atrium attached to inter-atrial septum at foramen oval level was detected (Figure 1).

**Figure 2:**
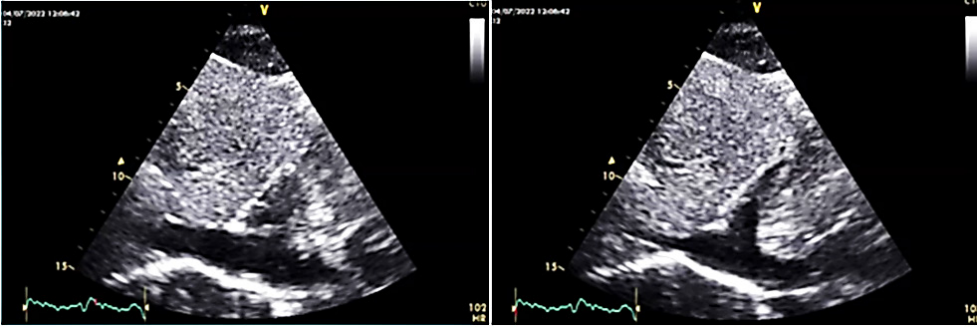
cyclic IVC dilation (because of outflow obstruction) during inspiration (left) and expiration (right)

 The mass was none homogeneous and several filamentous apparatus was detectable on mass’s surface on echo. In cardiac cycle intermittent protrusion of mass into the TV and dynamic TV stenosis results in cyclic IVC dilation (Figure 2).

 This was an important sign to confirm the correlation of LOC and this large mass.

 All data above was consistent with cardiac myxoma. Because a very dangerous anatomy of mass and history of episode of loss of consciousness, the next plan was to excision of mass by median

 sternotomy. Cardiac surgery by transfusion of pre and intra operative platelet was done.

**Figure 3:**
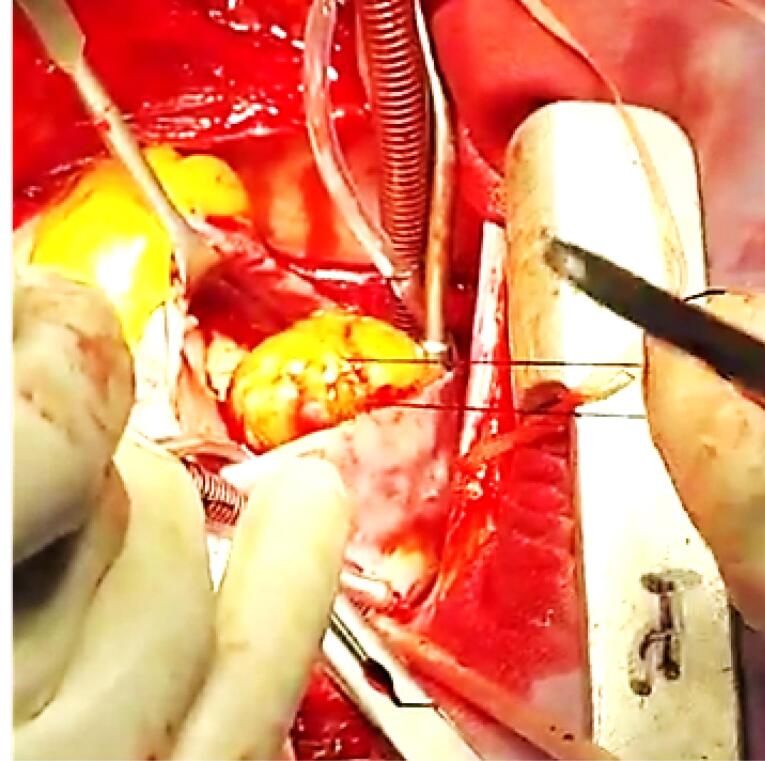
tumor excision with median sternotomy

 The surgery has been done and macroscopic findings confirmed the probable myxoma diagnosis (Figure 3). Post operation care was very good and patient was discharged without any complication, recommended for follow up. Patient was asymptomatic and episodes of loss of consciousness was disappeared on laboratory data the hemoglobin count was about 11 gr/dl and platelet count was increased to 490000/cc and in pathology report of mass cardiac myxoma was confirmed. Now after 5 months of surgery the patient is doing well and follow-up echocardiography is acceptable.

## Discussion

 As we know cardiac myxoma is the most common primary cardiac neoplasm that ranges in size from 1 to 15 cm. cardiac myxoma are about 0.25% of all cardiac diseases, 80% of cardiac masses are benign and cardiac myxoma are about 50% of benign cardiac tumors. These tumors are frequently diagnosed in middle age in about a mean age of 53 years.^1^

 These tumors are mostly seen in females with ratio of 3:1.^2^ sometimes diagnosis is difficult because of wide range of symptoms from asymptomatic patient to life threatening cardiac complication.^2^ In a case report by lee et al they reported a long period between start o symptoms and final diagnosis of myxoma for about 5 years.^1^

 The best diagnostic device for these tumors are echocardiography but chest computed tomography scan and cardiac magnetic resonance imaging are useful and may provide more information about cardiac tumors.^3^ Patients with myxoma develop cardiovascular symptoms (67%), systemic embolization (29%) constitutional symptoms (34%) including fever (20%), anorexia (18%).^4^

 About 37% of patients shows anemia, high ESR level, high CRP level.^5,6^ Despite some cases of thrombocytopenia have been reported, thrombocytopenia is a rare finding in cardiac myxoma.

 Treatment of these tumors are excision and resection of the mass because of the risk of embolization or cardiovascular complications.^1^ Some studies have reported in operative mortality rate of < 5%.^5,6^ To the best of our knowledge only 8 cases of thrombocytopenia and concomitant cardiac myxoma has been reported before,^7^ in addition originating of tumor from right atrium makes our case a more rare case.

 the mechanism of thrombocytopenia is not clear yet but some studies has shown breakdown of platelets caused by tumor induced outflow obstruction^8^ and this probable reason is encouraged and supported by rise in platelet count after removal of cardiac tumors.^9^ Another study showed that myxoma expresses CD 31 (also known as platelet endothelial cell adhesion molecule-1) which is known to change platelet level.^10^ Another cause is high levels of IL-6 (a pro-inflammatory cytokine) involved in many autoimmune diseases that can trigger an autoimmune loop eventually leading to immune thrombocytopenia ^11^. another probable cause is that myxoma exhibited microscopic bleeding, thrombosis and hemosidrosis which may consume platelets.^10^

 Another interesting issue in this article is the polycythemia in our patient before surgery (hgb:19.6gr/dl) that has been resolved after surgery (hgb:11gr/dl). Burns *et al* showed that the serum erythropoietin obtained from myxoma chamber during pre-operative cardiac catheterization was twice those of 4 control patients (250 MIU vs. 131 MIU); in which we can conclude the secretion of erythropoietin from myxoma tumor.^12^

## Conclusion

 In our case increase of platelet count after excision of tumor showed the relationship between cardiac myxoma and thrombocytopenia.

## Competing Interests

 The authors declare no conflict of interest financial or otherwise.

## Ethical Approval

 The ethics committee of Babol University of Medical Sciences, Iran (Approval number: IR.MUBABOL.REC.1401.171), approved the study.
